# Extending Brain-Training to the Affective Domain: Increasing Cognitive and Affective Executive Control through Emotional Working Memory Training

**DOI:** 10.1371/journal.pone.0024372

**Published:** 2011-09-19

**Authors:** Susanne Schweizer, Adam Hampshire, Tim Dalgleish

**Affiliations:** 1 Medical Research Council Cognition and Brain Sciences Unit, Cambridge, United Kingdom; 2 The Center for Brain and Mind, University of Western Ontario, Ontario, Canada; Royal Holloway, University of London, United Kingdom

## Abstract

So-called ‘brain-training’ programs are a huge commercial success. However, empirical evidence regarding their effectiveness and generalizability remains equivocal. This study investigated whether brain-training (working memory [WM] training) improves cognitive functions beyond the training task (transfer effects), especially regarding the control of emotional material since it constitutes much of the information we process daily. Forty-five participants received WM training using either emotional or neutral material, or an undemanding control task. WM training, regardless of training material, led to transfer gains on another WM task and in fluid intelligence. However, only brain-training with emotional material yielded transferable gains to improved control over affective information on an emotional Stroop task. The data support the reality of transferable benefits of demanding WM training and suggest that transferable gains across to affective contexts require training with material congruent to those contexts. These findings constitute preliminary evidence that intensive cognitively demanding brain-training can improve not only our abstract problem-solving capacity, but also ameliorate cognitive control processes (e.g. decision-making) in our daily emotive environments.

## Introduction

Imagine that on your way home you see a magazine advertising a brain-training program that promises to augment your capacity to mentally control how you process both neutral and emotional/personal information. The promised psychological benefits of the program include: (i) increased working memory (WM) capacity to actively maintain selected bits of information in the presence of salient distractors; (ii) increased fluid intelligence (G*f*) which would improve your abstract reasoning and problem-solving abilities; and (iii) greater control over the emotional/personal material you want to disengage from (e.g., disinterested members of an audience during a presentation) or engage with (e.g., interested listeners). Would you sign up?

Such a scenario is increasingly realistic, with cognitive training programs, colloquially known as ‘brain training’, that make similar promises becoming more available and commercially successful. However, few issues elicit more controversy within cognitive neuroscience than the question of whether transferable gains in cognitive functioning can be accrued through the regular practice of cognitive training tasks. The theoretical rationale for brain training is simple and straightforward. Neuroscientific research has identified neural networks that are commonly implicated in a wide range of cognitive tasks, including those purported to measure domain-general capacities such as G*f* [1]–[3]. Consequently, systematic training on a given task that reliably recruits these brain regions should not only improve performance on the training task but should also lead to transferable gains in performance on tasks that are dependent on the same neural substrates. Unfortunately, the empirical data have frequently failed to support this thesis. For example, recently, a high profile study involving more than 11,000 participants found no evidence for transferable benefits from tasks tapping reasoning, memory, planning, visuospatial skills and attention [4].

However, there is a small handful of studies that do report evidence of cognitive transfer effects following systematic computerized training in aspects of executive control in both patients (e.g., ADHD, schizophrenia) and healthy adults [5]–[8]. Perhaps the most persuasive of these is Jaeggi et al.'s [6] demonstration that systematic training on a complex WM task – a dual *n*-back task – accrues transferable benefits in G*f*, over and above any gains in WM capacity (indexed by an increase in digit span). This finding merits particular attention because G*f* has traditionally been viewed as highly heritable and stable [9], [10] and is positively correlated with a large number of desirable outcomes including academic success, and neurological, psychological and physical health [11]–[14]. Evidence showing change in G*f* through WM training, therefore, has far reaching implications.

Why have studies such as that of Jaeggi et al. [6] realized positive transfer effects from cognitive training, while the majority of investigations have struggled to find any support for transferable benefits of training? Critics of the cognitive training endeavor would argue that such intermittent significant results may simply be false positives (e.g., [15]) and that the jury should remain out, pending replication of any given findings by an independent research team. An alternative view, however, is that studies with positive findings have taken care to ensure that the training regime imposes heavy and multiple executive demands on the trainee that increase in tandem with improved performance, such that the trainee is always operating at maximum capacity and opportunities for the development of automatic processes and task-specific strategies are minimized.

Given the fundamental importance of elucidating whether brain training can lead to transferable gains in G*f*, the first aim of the present study was to investigate the replicability of transfer effects from extensive training on a highly demanding dual *n*-back WM task to improvements in performance on a non-trained measure of short-term/WM – digit span – and on G*f* over and above digit span.

Of course, transferability across cognitive tasks represents only one form of transfer. For any training methodology to have a wide-ranging impact on real-world cognition, there also needs to be transfer across training *content*. In particular, as our hypothetical advertisement suggests, many cognitive challenges in day-to-day life require the monitoring, manipulation and inhibition of information that is either personally relevant or emotionally-laden and often both. Yet, the discourse surrounding the promises of brain-training has so far been limited to the investigation of transfer effects onto cognitive performance that requires the manipulation of only neutral material.

A wealth of evidence on a wide range of tasks (e.g., [16]–[18]) suggests that the salience of such affective information interferes with our capacity to subject it to executive control. A prototypical example is the emotional Stroop task [19]–[21]. There are variants of this paradigm. But the task always requires stimuli to be processed on two key components, whereby participants respond as quickly as possible to one component (the target) while ignoring the other (the distracter). When both these components are affective (e.g., an emotional word and facial expression), the processing of the target component is reliably facilitated if they share affective valence (congruent trials), but impaired when the distracter component is of a different valence (incongruent trials), relative to a control condition when the distracter is neutral (e.g., [22]). The key question then is whether cognitive training on tasks presenting only neutral information can accrue transferable benefits in terms of cognitive processing of emotionally salient or personally relevant material, or whether a training context populated with such material is required. Surprisingly, this issue, which is central to the ecological utility of any training effects and to any widespread appeal of brain-training, remains unexamined in the literature, and addressing it was the second aim of the current study.

To this end, we modified the dual *n*-back task that had successfully induced transfer effects onto non-trained WM tasks and G*f* in the aforementioned Jaeggi et al. [6] study. While the original training presented participants with letters and squares in the auditory and visuospatial modalities, we presented words (over headphones) and faces (appearing in one of 16 possible locations on a 4×4 grid).

**Figure 1:**
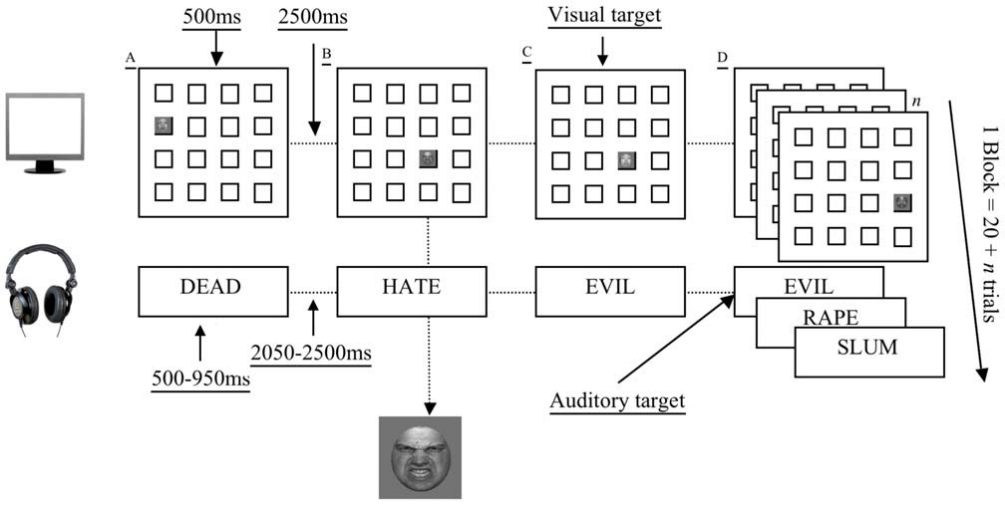
Emotional dual n-back block (*n* = 1). The figure depicts a block of the emotional version of the dual *n*-back task (training task) where *n* = 1. The top row shows the sequence across trials (A, B, C, D, etc.) of visually presented stimuli in a 4×4 grid (the visual stimuli were presented on a standard 1280×1024 pixel computer display). A picture of a face appeared in one of the 16 possible grid positions on each trial. Simultaneously, with the presentation of these visual stimuli on the computer display, participants heard words over headphones (second row in the figure). Participants were required to indicate, by button press, whether the trial was a ‘target trial’ or not. Targets could be visual or auditory. In the example here, Trial C is a visual target. That is, the face in Trial C is presented in the same location as the face in Trial B (i.e., *n* = 1 positions back). Note, the faces are of different actors. For visual stimuli participants were asked to ignore the content of the image and solely attend to the location in which the images were presented. In the current example, Trial D was an auditory target trial because ‘Evil’ is the same word as the word presented in Trial C - *n* positions back (where *n* = 1). Each block consisted of 20+*n* trials.

We then compiled two versions of the task. The first involved hearing neutral words and seeing neutral faces, the second version involved highly emotional words (e.g., rape) and faces with negative expressions (see Figure 1). In addition, we designed a third training paradigm – a non-WM-dependent feature matching control training. The rationale for this third task was to provide a control condition to take account of any placebo effects of receiving ‘brain-training’. This design enabled us potentially to replicate Jaeggi et al.'s [6] impressive effects of a dual *n*-back training program on WM and G*f* relative to an *active* control group.

Our first hypothesis then was that training on the dual *n*-back task (irrespective of the valence of the content), relative to control task training, would lead to transferable gains in short-term memory/WM capacity (measured by digit span) and in G*f* (measured by Raven's Progressive Matrices) over and above any gains in digit span.

Our second hypothesis was that transferable gains in affective executive control would only accrue for those undergoing dual *n*-back training with affectively laden stimuli. To evaluate this we selected the emotional Stroop paradigm described earlier as the target transfer task, as it is well established in the literature as an index of affective executive control and allows us to examine both facilitation and interference effects associated with affective stimuli.

## Results

### Training performance

**Figure 2:**
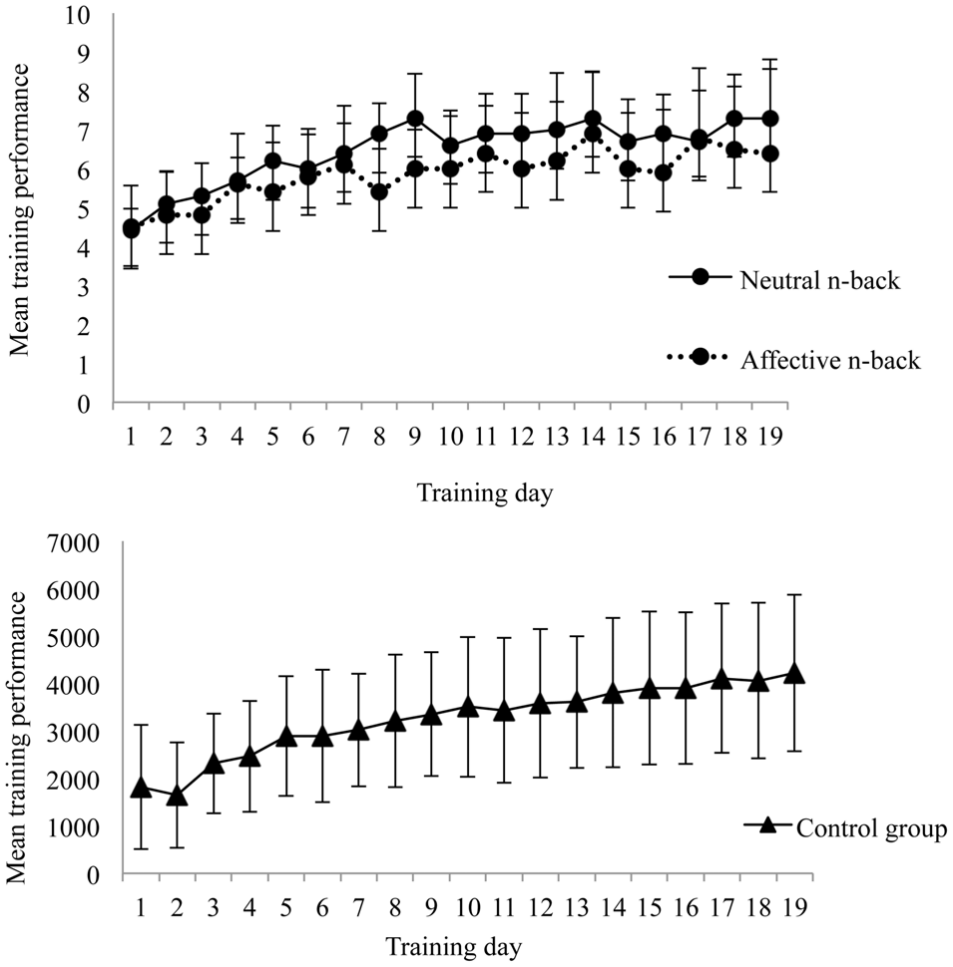
Performance improvement across training days. These two graphs show the linear improvement in average training performance across training days for all three groups. For the neutral *n*-back and affective *n*-back the score refers to mean level of *n*-back achieved. The score on the feature match task for the control group constitutes a mean composite score (see Methods and Materials).

Analyses of the training functions revealed that both *n*-back trained groups and controls showed a linear improvement on their respective training tasks as a function of time (Linear contrast: *F_Control_*(1, 15) = 39.93, *P*≤0.001; *F_Neutral_*(1, 14) = 10.21, *P* = 0 .008; *F_Affective_*(1, 13) = 17.29, *P* = 0.002; Figure 2). As expected, the *n*-back groups showed a significantly greater performance increase on the tasks they trained with, relative to the controls: neutral *n*-back *F*(1, 42) = 9.92, *P*<0.001, η_p_^2^ = 0.42; affective *n*-back *F*(1, 42) = 15.84, *P*<0.001, η_p_^2^ = 0.43. Performance of the two *n*-back groups pre- to post- training did not differ significantly on either the neutral *F*(1, 27) = 1.02, *P*>0.05 or affective *F* (1, 27)<1 *n*-back tasks. Similarly, the control group showed a significantly greater pre- to post-training improvement on the feature match task they trained on, compared with the *n*-back groups *F*(1, 42) = 41.09, *P*<0.001, η_p_^2^ = 0.67.

### Cognitive transfer effects

To demonstrate transfer effects it is first necessary to show a pre- to post-training improvement on the transfer task in the trained group. It is then necessary to demonstrate that this improvement is related to training by showing that the increment is significantly greater than any change in control participants. As anticipated, there were no significant differences in the magnitude of any cognitive transfer effects between the two active training groups (neutral and affective), *F*s<1. Therefore, data were pooled across the two n-back groups into a larger ‘Training Group’ for analyses of cognitive transfer.

On our measure of short-term/WM – digit span – there were no significant differences between the control and training groups at pre-training *F*(1, 43) = 2.89, *P* = 0.10 (see Table S1). As predicted, participants in the training group showed a significant improvement on digit span *F*(1, 28) = 33.96, *P<*0.001, η_p_^2^ = 0.55. However, this was not true of controls *F*(1, 15) = 1.89, *P* = 0.19, η_p_^2^ = 0.11, and the gain was significantly greater in the training group participants compared to controls *F*(1,43) = 5.92, *P* = 0.02, η_p_^2^ = 0.12.

**Figure 3:**
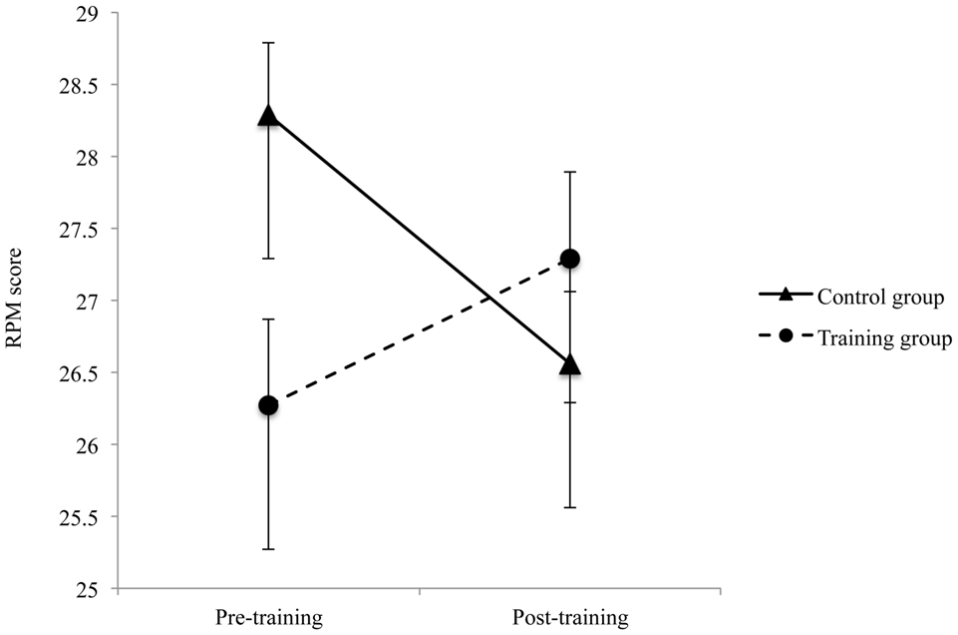
Transfer benefits of training to fluid intelligence. Figure 3 reports mean group scores achieved on the Raven's Progressive Matrices (RPM; measure of fluid intelligence). Means (standard deviation) for the control and *n*-back trained groups are 28.29 (0.64), 26.56 (0.46) at pre-training and 26.27 (0.73), 27.29 (0.53) at post-training, respectively.

We next examined transfer to G*f* (see Figure 3). As G*f* tests include a substantive WM component, Jaeggi et al. have argued that it is important to demonstrate transfer to G*f* over and beyond training-induced improvements in WM as indexed by digit span (for a further discussion see Jaeggi et al. [6]). Consequently, our analysis of G*f* effects covaried out gains in digit span to mirror Jaeggi et al.'s approach directly. Replicating their results, we found a significant gain in G*f* scores in the training group over and above gains on the digit span task *F*(1, 26) = 3.00, *P* = 0.05, η_p_^2^ = 0.10. In contrast, the control group showed a non-significant decrease in G*f*, F<1, and the critical group by time interaction was significant, *F*(1, 40) = 7.47, *P* = 0.01, η_p_^2^ = 0.16. As can be seen in Figure 3, there was a trend toward a significant group difference in G*f* (RPM scores) at pre-training, *p*≤0.10. This raises the possibility that the relative gains in G*f* in the training versus control groups may be to some extent an artefact of baseline differences. However, the interactive effect of transfer as a function of group remained significant even after more closely matching the training and control groups for pre-training RPM scores (by removing the highest scoring controls) *F*(1, 30) = 3.66, *P* = 0.032, η_p_^2^ = 0.10. The adjusted means (standard deviations) for the control and training groups were now 27.20 (1.93), 26.63 (2.60) at pre-training (*t*(43) = 1.29, *P*>0.05) and 26.50 (4.50), 27.07 (2.16) at post-training, respectively. Moreover, there was a trend for the gain in G*f* to be positively correlated with improvements in *n*-back performance across training *r*_(29)_ = 0.36 at *P* = 0.057, suggesting that such gains were indeed a function of training.

### Affective transfer effects

**Figure 4:**
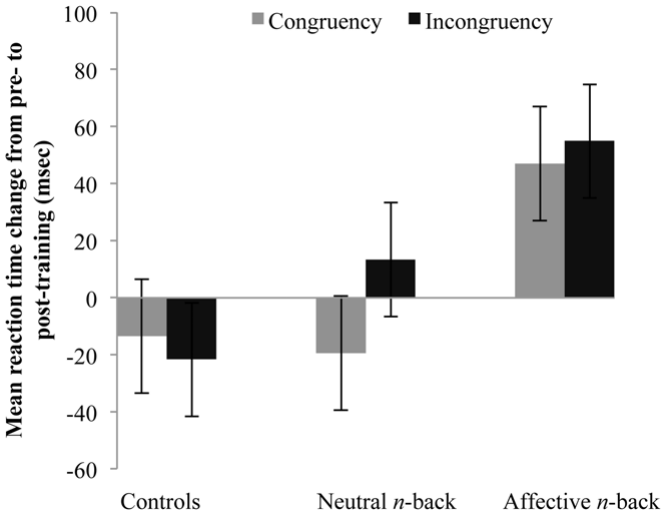
Training benefits on the emotional Stroop only in affective training group. Changes in reaction time (post-training reaction time in msec – pre-training reaction time in msec) in congruent and incongruent indices on the emotional Stroop task from pre-training to post-training, displayed such that the higher the change the greater the training-related benefits.

Affective transfer was conceptualized as pre- to post-training gains on the emotional Stroop task, measured with both a congruency index (reduced reaction time latencies for congruent trials where the distractor face and target word depict the same emotion) and an incongruency index (reduced reaction times for incongruent trials, where a distracting face depicts an emotion incongruent with the emotion of the target word) (see Methods section). We predicted that any effects of affective transfer would be differentially greater on both indices in the affective training group. Consequently, affective transfer analyses included all three groups. To examine affective transfer we used a multivariate analysis of variance (MANOVA) with both the congruency and incongruency indices as the dependent variables. The results showed that for the affective *n*-back trained participants there were significant pre- to post-training improvements in overall emotional Stroop performance *F*(2, 12) = 4.02, *P* = 0.02, η_p_^2^ = 0.40 (see Figure 4). The univariate output revealed significant gains in the affective training group for both incongruent, *F*(1,13) = 7.50, *P* = 0.009, η_p_^2^ = 0.37, and congruent indices, *F*(1,13) = 4.76, *P* = 0.024, η_p_^2^ = 0.27. These data provide clear support for affective transfer effects following affective *n*-back training. In contrast, there was no evidence supporting affective transfer effects in either the neutral *n*-back trained group or the controls, all multivariate and univariate Fs<1.24, Ps>0.4 (see Figure 4).

In the critical comparisons across the three groups, the MANOVA revealed that the overall group by time multivariate interaction was significant, *F*(2, 39) = 4.37, *P* = 0.02, η_p_^2^ = 0.18. Deconstructing this effect within the omnibus MANOVA revealed that the affective *n*-back trained group showed significantly greater transfer effects to the emotional Stroop task than both the control group, congruency: *F*(2, 39) = 4.27, *P* = 0.04, η_p_^2^ = 0.14; incongruency: *F*(2, 39) = 6.75, *P* = 0.01, η_p_^2^ = 0.19; and the neutral *n*-back group, congruency: *F*(2, 39) = 4.95, *P* = 0.03, η_p_^2^ = 0.16; incongruency: *F*(2, 39) = 1.95, *P* = 0.08, η_p_^2^ = 0.08, although the latter incongruency effect was at trend level (see Table S2 for mean reaction times at pre- and post-training for all groups). However, the neutral *n*-back training and control groups did not differ from each other, incongruent: *F*<1, congruent: *F*(1, 27) = 2.96, *P* = 0.10, η_p_^2^ = 0.10.

## Discussion

Our findings provide some support for the reality of both training and transfer effects as a function of executively demanding dual-task WM training. We showed that practice makes you better on the task you train on, in that our participants' performance on their respective training tasks improved as a linear function of time spent training. The groups who trained on the dual *n*-back tasks also improved on an untrained WM transfer task, the digit span, which replicates previous findings [6], [8], [23]. No significant digit span transfer was evident in controls who trained on a non-WM demanding feature match task. The digit span is a measure of attentional control that seems to depend on the recruitment of similar neural substrates as the *n*-back task and to be dependent on shared cognitive processes [24]–[26]. This transfer is particularly noteworthy in the light of Owen et al.'s [4] data showing no transfer effects to digit span following repeated training on various less demanding ‘brain-training’ tasks. It is possible that continuously training at maximum performance level (i.e., sustained effortful training) may partially account for this finding. However, both the control group in the present study and the participants in Owen et al.'s study [4] had the opportunity to keep their training challenging by augmenting response rates. Additionally, some of the tasks used by Owen et al. [4] were titrated to participants' performance levels. Yet our participants gained on average in excess of two digits increase in span over the course of 20 training sessions, a feat that Owen et al. [4] inferred would take their subjects over four years of training. An alternative explanation then lies in the unique task properties of the dual *n*-back task used here compared to the control task and to other brain training tasks. Specifically, the dual *n*-back task places exceptionally high demands on executive control processes, in particular by requiring manipulation of bimodal stimulus material. As Jaeggi et al. [6] note, this provides only minimal opportunity for process automatization and the development of task-specific strategies.

Such continuous engagement of executive processes, especially WM, during training has also been proposed to account for the transfer gains to G*f* [6], [23], [27]. G*f* appears to be cognitively dependent on faculties such as abstract reasoning and problem solving in addition to executive control, and the underlying neural substrates appear to be shared [23], [28], [29]. Our data provide some support for this by showing significant pre- to post-training improvements in G*f*, over and above any gains in WM indexed by digit span, in participants who trained on the dual *n*-back task, but not in our control participants who received equal amounts of training on a non-WM demanding feature match task. It seems plausible that training-related gains in the efficiency of these neural networks are driving the observed transfer effects onto G*f* and a challenge for future research is to elucidate the neural substrates of such training and transfer effects. Irrespective of the underlying mechanisms, the current results which further support the malleability of G*f* to training have a potentially wide range of (encouraging) implications for educational, neuropsychological and psychopathology treatment settings, if they prove to be robust.

In addition to this important support of cognitive transfer effects, we extended transfer into the affective domain. Specifically, we showed that only training on the dual *n*-back task using affective stimuli accrued transfer gains on the emotional Stroop task – a standard measure of affective executive control. Importantly, there was no support for such affective transfer effects in participants who trained on the dual *n*-back task with neutral stimuli or in controls who trained on the feature match task.

These findings suggest that individuals can learn through training on a task that improves executive control of affective material to subsequently manipulate emotional information in other settings more successfully. Participants got better at engaging with goal-relevant affective material, while ignoring highly emotional material that is not pertinent or may distract from the target task. Specifically, they learnt to disengage from task-irrelevant material in the visuospatial task where the facial emotion provides no information for task performance, while selectively attending to emotional information in the auditory modality of the task where the emotional word is the task-relevant stimulus. The same dissociating capacity is required in the emotional Stroop task for incongruent and congruent trials. Translating this into everyday life, the implication is that such training may improve participants' decision making in situations that require the manipulation of emotional material. Moreover, patients with emotional disorders that are characterized by difficulties in exerting cognitive control in order to selectively engage and disengage from affective information may benefit from such training (e.g., depression: [30], [31]; anxiety disorders: [32], [33]).

This preliminary finding that affective transfer effects are selective to affective executive training is important considering that most of the research effort in terms of training in the fields of neuropsychology, emotional psychopathology, and decision making is conducted with emotionally neutral stimuli. Such studies relying solely on neutral material may arguably fail to target processes specific to the manipulation and processing of affective information. Again, a challenge for future research is to identify the shared cognitive and neural substrates that mediate the transfer of affective executive control and how these differ from the cognitive transfer effects described above.

There are some potential caveats regarding the current results that merit discussion. Firstly, the sample sizes were modest due to the large demands on time and resources placed on participants. Future studies should aim for larger scale replications. Secondly, our study used a high-functioning and intelligent student sample. While there is no evidence to suggest that baseline intelligence test scores moderate training gains (e.g., [6]), it is possible that some of the transfer effects in the present data were diminished due to ceiling effects. Moreover, our groups showed a trend for pre-training differences in G*f*. Although the G*f* transferable gains we found appear to be somewhat related to training gains and the effects remain when we trim the groups to provide a better match for pre-training G*f*, it is important to note that some degree of regression to the mean may be influencing the results. Finally, we did not include a control group that did not receive active WM training but was nevertheless exposed to emotional material during training. This would have allowed us to rule out the unlikely possibility that affective transfer gains were simply a function of mere exposure to emotional information during training.

In conclusion, our study supports the effectiveness of highly demanding dual *n*-back training in increasing WM capacity on different tasks and in increasing G*f*. These findings, alongside those of others (e.g., [6]), suggest that such training programs could be employed to improve executive control and G*f* in individuals with executive control deficits to alleviate symptomatology associated with deficient executive control. We also provide preliminary support for the notion that training in an affective context uniquely can accrue transferrable benefits in affective executive control on a separate task assessing goal-directed (dis)engagement with emotional material. Affective WM training of this nature, unlike training with purely neutral materials, thus has the potential to benefit recipients' everyday decision making which very frequently involves material of an affective or personally salient nature, as well as to address core pathological processes in emotional disorders.

## Methods

The study and consent procedure were approved by the Cambridge Psychology Research Ethics Committee. Prior to the study prospective participants were provided with detailed written and oral information about the study. Participants who chose to participate signed a written informed consent form.

### Participants and Procedures

Forty-five participants (28 female; *M* age = 25 years; range: 21–30 years) were recruited through a University of Cambridge student bulletin, and randomly assigned across three different training conditions: the control training (*n* = 16); the neutral dual *n*-back training (*n* = 14); and the affective dual *n*-back training (*n* = 15). All participants were tested in two two-hour-long sessions, one on each of two consecutive days, at both pre- and post-training (with time of day at pre- and post-training held constant). The first post-training session took place the day after the training had ended. Participants trained for twenty days in four five-day blocks followed by two rest days. The control training duration was fixed to 20 minutes per day, whereas duration varied between 20–30 minutes for the dual *n*-back training groups (depending on the level of *n*-back the participants reached in a given session). Participants needed to complete at least 75% (15 days) of the training. The groups did not differ on the number of training days completed (*M_Control_* = 18.67; *M_Neutral_* = 19.00; *M_Affective_* = 18.00, *F*(1,42) = 1.91, *P* = 0.16). Moreover, the training and control groups did not significantly differ in demographic characteristics including age, gender, education and race (see Table 1).

**Table 1:** Group demographic information.

	Control training (*n* = 16)	Affective *n*-back training (*n* = 15)	Neutral *n*-back training (*n* = 14)	*F*	Х^2^	*p*
Age (*M* (sd))	25 (2.70)	25 (2.69)	25 (2.01)	0.26		0.77
Gender (*n*)	9	10	8		0.41	0.82
Education (*M* (sd))	3.60 (0.51)	3.33 (0.98)	3.43 (0.51)	0.55		0.58
Race (*n*)	10/5/1	13/1/1	9/5/0		2.18	0.34

The table represents the three groups' demographic characteristics. Age (*M* (sd)): Mean and standard deviation of age in years; Gender (*n*): number of women; Education (*n*): 1 - 11^th^ grade/2 - High school /3 - Graduate/4 - Postgraduate; Race (*n*): Caucasian/Asian/mixed.

### Materials

#### The dual n-back training task

The dual *n*-back training task was modeled on the task designed by Jaeggi et al. [6]. In our modified version, participants were simultaneously presented with images of faces that appeared for 500 ms in one of 16 locations on a 4×4 grid on the computer screen, and words (duration 350–900 ms) over binaural headphones (see Figure 1). Each combined face-word presentation was followed by a 2500 ms interval during which the grid was blank and there was no sound. During this interstimulus interval participants responded via key press (left/right arrow keys respectively for face location and word) if either the face or word stimuli from the current trial matched the corresponding face or word stimuli presented *n*-positions back. Participants therefore needed to remember the stimuli *n* positions back whilst monitoring both modalities for each new trial.

Each training session consisted of twenty blocks of 20+*n* trials (i.e., picture-word pairs). There were six target trials per modality in each block. The training always started at *n* equals one. If three or more consecutive trials were completed accurately the level of *n*-back increased by one on the next block. Conversely, if five or more successive trials were completed inaccurately the level of *n*-back decreased by 1 on the next block, to a minimum of *n* = 1. By these means the task was titrated so participants continuously operated at their maximum performance level. In the neutral condition the stimuli (i.e., faces and words) were neutral in valence and they were emotional in valence in the affective condition. For more information about the stimulus material see Text S1.

#### Control training task

The feature match task presented participants with two panels. Each panel contained a minimum of eight shapes. Participants needed to indicate whether the panels were made up of identical shapes. The numbers of shapes presented in the panel increased with performance but were limited to 12 during the training phase. The outcome score was a composite score of the number of correct trials, number of trials attempted and reaction time.

#### Cognitive transfer tasks

We selected the same cognitive transfer tasks as used by Jaeggi et al. [6] for the purpose of independent replicability. The forward digit span test requires participants to recall digits that were read out loud to them in the order that they were presented. A participant's span was the maximum number of digits participants recalled without error. The digit span is a widely used measure of WM [34]. We assessed G*f* with the Raven's Progressive Matrices (RPM; [35]) – a standard measure in the literature. Each RPM item presented participants with a matrix of visual patterns with one pattern missing. The participant chose how the matrix should be completed by selecting a pattern from a series of alternatives. We used parallel versions of the RPM (even and uneven numbered pages), which we counterbalanced across participants and pre- and post-training. The RPM is scored on a scale from 0–30, with each correct matrix earning participants one point.

#### Affective transfer task

Participants' ability to automatically inhibit interference from affectively valenced stimuli was assessed with a version of the emotional Stroop task developed by Preston and Stansfield [22]. The task required participants to categorize an affective adjective as related to one of three emotions (angry, happy or sad), while ignoring the valence of the expression on a face upon which the adjective was superimposed. The presentation-rate of the stimuli was self-paced (for a detailed task description see Preston and Stansfield [22]). The emotional Stroop task generates two indices of affective executive control: the *incongruency index* is the cost in reaction time to correctly categorize an emotional adjective when the background face depicts an incongruent emotional expression relative to when the face depicts a neutral emotional expression. The *congruency index* reflects the facilitation in reaction time to categorize an emotional adjective when the background face depicts a congruent facial expression relative to the neutral condition. Training transfer effects on the task were depicted as decreases in the incongruency cost and increases in the congruency facilitation effects from pre- to post-training.

## Supporting Information

Text S1 **Stimulus material for the affective and neutral versions of the dual n-back training tasks.**
(DOC)

Table S1 **Mean pre- and post-training scores on the digit span task for the combined training group and control group.** *Note.* Pre-*M*: mean digit span at pre-training; Post-*M*: mean digit span at post-training; *sd*: standard deviation.
(DOC)

Table S2 **Mean pre- and post-training reaction times on the Emotional Stroop across the three training groups.** *Note.* Pre-*M*: mean reaction time at pre-training; Post-*M*: mean reaction time at post-training; *sd*: standard deviation. The means and standard deviations are reported in msec.
(DOC)
