# Supplementary material for: Extending Brain-Training to the Affective Domain: Increasing Cognitive and Affective Executive Control through Emotional Working Memory Training
Source: PLoS One. 2011 Sep 19;6(9):e24372. doi: 10.1371/journal.pone.0024372 (PMC3176229; doi:10.1371/journal.pone.0024372)
Supplement: Text S1 — Stimulus material for the affective and neutral versions of the dual n-back training tasks. (DOC) [file pone.0024372.s001.doc]

Text S1: *Stimulus material for the affective and neutral versions of the dual n-back training tasks.* Each training session presented participants with a unique set of combinations of twenty different faces and ten different words. The faces set constituted pictures from 4 individuals (2 female, varying races). Pictures from two models (1 female) were randomly presented across blocks. Half the words from each set were recorded by a woman and the other half by a man. Both were native British English speakers without regional accent. For the visuospatially presented stimuli (i.e., faces presented in a 4x4 grid) participants were required to respond to the location of the face rather than the face itself. The faces presented were taken from the NimStim Face stimuli set and the Karolinska Directed Emotion Faces database (1-2). The faces were all placed into an oval on a 380 pixels x 380 pixels grey mask. The auditory stimuli were taken from the Affective Norms for English Words (ANEW) database (3). All stimuli were presented in Adobe Flash. In the neutral training all face stimuli had neutral expressions and words were neutral, the affective dual *n*-back group was presented with face stimuli with sad, fearful, angry, and disgusted expressions and the words were negative.

References

[1] Lundqvist D, Flykt A Öhman A (1998) The Karolinska Directed Emotional Faces - KDEF, CD ROM from Department of Clinical Neuroscience, Psychology Section, Karolinska Institutet, ISBN 91-630-7164-9.

[2] Tottenham N, Tanaka JW, Leon, AC, McCarry T, Nurse M, Hare TA et al. (2009) The NimStim set of facial expressions: judgments from untrained research participants. Psych Res 168: 242-249.

[3] Bradley BP, Lang PJ (1999). Affective Norms for English Words (ANEW): Instruction manual and affective ratings. Technical report C-1, Gainesville FL: The Center for Research Psychophysiology, University of Florida.
