# Supplementary material for: Extending Brain-Training to the Affective Domain: Increasing Cognitive and Affective Executive Control through Emotional Working Memory Training
Source: PLoS One. 2011 Sep 19;6(9):e24372. doi: 10.1371/journal.pone.0024372 (PMC3176229; doi:10.1371/journal.pone.0024372)
Supplement: Table S1 — Mean pre- and post-training scores on the digit span task for the combined training group and control group. Note. Pre-M: mean digit span at pre-training; Post-M: mean digit span at post-training; sd: standard deviation. (DOC) [file pone.0024372.s002.doc]

Table S1. Mean pre- and post-training scores on the digit span task for the combined training group and control group

|  | Control (*n*=16) | | Neutral training (*n*=14) | | | Emotional training (*n*=15) | |
| --- | --- | --- | --- | --- | --- | --- | --- |
|  | Pre-*M* (sd) | Post-*M* (sd) | Pre-*M* (sd) | Post-*M* (sd) | | Pre-*M* (sd) | Post-*M* (sd) |
| Digit span | 11.31 (2.77) | 11.93 (2.89) | 11.00 (3.01) | | 12.79 (2.39) | 10.20 (2.57) | 12.74 (2.56) |
