# Supplementary material for: Extending Brain-Training to the Affective Domain: Increasing Cognitive and Affective Executive Control through Emotional Working Memory Training
Source: PLoS One. 2011 Sep 19;6(9):e24372. doi: 10.1371/journal.pone.0024372 (PMC3176229; doi:10.1371/journal.pone.0024372)
Supplement: Table S2 — Mean pre- and post-training reaction times on the Emotional Stroop across the three training groups. Note. Pre-M: mean reaction time at pre-training; Post-M: mean reaction time at post-training; sd: standard deviation. The means and standard deviations are reported in msec. (DOC) [file pone.0024372.s003.doc]

Table S2. Mean pre- and post-training reaction times on the Emotional Stroop across the three training groups

|  | Controls (*n*=16) | | Neutral training (*n*=14) | | Emotional training (*n*=15) | |
| --- | --- | --- | --- | --- | --- | --- |
|  | Pre-*M* (sd) | Post-*M* (sd) | Pre-*M* (sd) | Post-*M* (sd) | Pre-*M* (sd) | Post-*M* (sd) |
| Neutral | 978.34 (72.92) | 901.58 (44.24) | 901.99 (48.06) | 855.78 (52.33) | 972.83 (56.29) | 893.34 (42.81) |
| Congruent | 955.83 (62.21) | 886.03 (51.70) | 913.77(62.21) | 823.80 (47.81) | 922.11 (50.34) | 865.88 (44.91) |
| Incongruent | 1001.11 (48.59) | 942.93 (38.15) | 970.35 (54.82) | 874.42 (49.80) | 998.36 (55.91) | 913.06 (48.04) |
